# Effects of Water Addition on Laminar Premixed Ethanol/Air Flame at Elevated Temperature and Pressure

**DOI:** 10.1021/acs.energyfuels.5c03872

**Published:** 2025-10-22

**Authors:** Linlin Yang, Xiaohang Fang, Felix Leach

**Affiliations:** † Department of Engineering Science, 6396 University of Oxford, Oxford OX1 3PJ, U.K.; ‡ Department of Mechanical and Manufacturing Engineering, Schulich School of Engineering, 2129 University of Calgary, Calgary T2L 1Y6, Canada

## Abstract

In this study, the effects of water addition on ethanol/air flames with high water content at elevated temperature and pressure are numerically investigated, and a novel correlation for their laminar burning velocity (LBV) is proposed based on experimental results. The dependence of the temperature and pressure exponents on thermodynamic parameters is numerically analyzed and considered in the new correlation to optimize the existing correlation. The fitting results of LBV correlations based on experimental measurements using a constant-volume method demonstrate that incorporating high-order and cross terms into the correlation enhances the overall performance, particularly under fuel-rich conditions where existing correlations exhibit significant discrepancies. The new LBV correlation of hydrous ethanol/air mixtures performs well over a wide range of elevated temperatures and pressures and agrees well with experimental data in the literature at high temperatures and pressures. The calculated LBV using the new correlation is also in good agreement with simulations using various mechanisms, except for fuel-rich mixtures with high water content, where the LBV is underpredicted by all mechanisms considered, suggesting further development of chemical mechanisms is needed. A sensitivity analysis suggests that under high water content, the dominant reactions of fuel-rich flames are different from those in stoichiometric and fuel-lean mixtures, highlighting that fuel-rich hydrous ethanol/air flames are very sensitive to water addition. The results also suggest that water addition leads to a reduction in the LBV. Both the burnt gas temperature and the peak heat release rate decrease with the water content, with a stronger influence on fuel-rich ethanol/air mixtures. Furthermore, the dilution effect of water addition constitutes the single largest effect in reducing the LBV, while chemical and thermophysical effects are found to be comparatively minor. The findings are helpful in understanding the fundamental combustion properties of hydrous ethanol and optimizing the LBV correlation under engine-relevant conditions.

## Graphical abstract

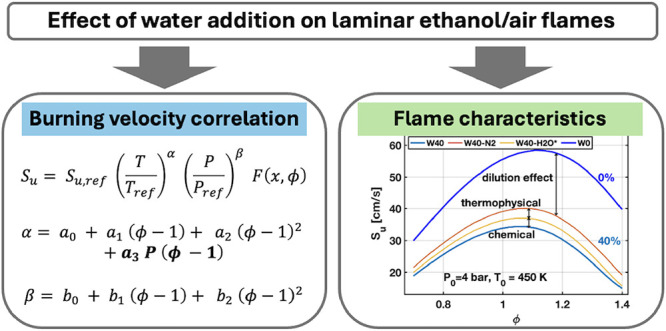

## Introduction

Due to increasing concerns about global warming and climate change, reducing the use of conventional fossil fuels has attracted much attention in the combustion research community. Ethanol is the most widely used biofuel and plays an important role in reducing the reliance on conventional fossil fuels.^1–4^ Ethanol is also commonly used as a blending fuel in gasoline.^5^ Therefore, it is of great interest to understand the combustion characteristics of ethanol and its blends in engines.^6–8^ In order to reduce pollutants, a water addition strategy can be employed to reduce the flame temperature, thereby reducing the formation of NO_*x*_. Water addition in ethanol also has the potential to suppress levels of knock, allowing for higher thermal efficiencies. On the other hand, some water content may be present in the ethanol since ethanol can easily absorb water during its production and utilization. To enable the direct use of hydrous ethanol, it is essential to understand its combustion characteristics. Additionally, the water content can influence key combustion phenomena such as the flashback, autoignition, and instabilities,^9^ which also highlights the need to investigate the effects of water addition on ethanol/air flames.

Laminar burning velocity (LBV) is one of the most important parameters of a fuel. It depends on the thermodynamic states of the mixture, including temperature, pressure, and mixture composition.^10^ LBV is crucial for the development and validation of detailed chemical mechanisms.^1,11,12^ In addition, it is a key parameter for numerical simulations and serves as a primary parameter in various combustion models.^13,14^ Therefore, accurate LBV data for hydrous ethanol are of great importance, particularly at elevated temperatures and pressures that are close to engine-relevant conditions. In practical engine simulations, empirical correlations are commonly used to calculate LBV due to their simplicity.^15^ In practical engine simulations, combustion models based on LBV correlations are more computationally efficient than those based on detailed chemistry. Typically, the LBV in a correlation is expressed as a power-law function of temperature and pressure.^16–19^ Therefore, the temperature and pressure exponents significantly affect the performance of correlations.^17,20^ Due to the difficulty in LBV measurement at high pressure, the correlations are often obtained at lower pressure and temperature. As a result, the form of correlation has a great influence on its performance at high temperature and pressure. To accurately represent the experimental results of LBV measurements obtained using the constant-volume method, it is necessary to determine an LBV correlation form with minimal error. Meanwhile, the existing correlation^17^ demonstrates scatter for fuel-rich ethanol/air flames with high water content, although its performance is good under fuel-lean or low dilution conditions. To reduce the errors in the LBV calculation introduced by the correlation form, it is important to examine the temperature and pressure exponents in the correlation when the water content is high, particularly at an elevated temperature and pressure.

The fitting and validation of LBV correlations require a large amount of experimental data over a wide range of thermodynamic conditions. In the literature, many studies have investigated the LBV of ethanol/air flames without water addition.^18,21–26^ For example, the experimental studies by Sileghem et al.^27^ and Konnov et al.^22^ measured the LBV of ethanol for a range of temperatures between 298 and 358 K at atmospheric pressure. These studies also analyzed the temperature dependence of LBV on the equivalence ratio and found that there exists a minimum in the temperature exponent, which offers useful information for the development of LBV correlations. Knorsch et al.^28^ measured the LBV of ethanol/air mixtures at temperatures up to 473 K using the heat flux method. A similar study by Katoch et al.,^29^ using an externally heated mesoscale diverging channel, measured the LBV of ethanol/air mixtures in the temperature range of 350–620 K at atmospheric pressure. For a very high initial temperature around 1000 K, Zheng et al.^30^ measured the LBV of ethanol in argon-based oxidizers using a shock tube. However, none of these experimental measurements provided LBV data at elevated pressures. The experimental study by Beeckmann et al.^24^ measured the LBV of ethanol/air flames at high pressures and temperatures using the spherically expanding flame method. This study showed that the LBVs from simulations are slower than those from experimental measurements at high pressure, demonstrating that the improvement of detailed chemical models is necessary for an accurate prediction of LBV. Additionally, Aghsaee et al.^31^ reported the LBV of ethanol/air mixtures at pressures up to 5 bar or temperatures up to 473 K using spherically expanding flames with the constant-pressure method. Moreover, a review by Konnov et al.^32^ showed that the measured LBV of ethanol/air mixture at atmospheric pressure agrees well with the numerical prediction using many chemical mechanisms, while there exists a significant discrepancy under fuel-rich conditions. This review also pointed out that experimental data of the LBV of ethanol/air flames at high temperature and pressure are required to improve the chemical mechanisms. The study by Hinton et al.^33^ reported the LBV of ethanol/air mixture at high temperatures and pressures. A 14-term power-law correlation was used to model the dependence of LBV on temperature, pressure, equivalence ratio, and the water volume fraction. However, the correlation proposed was found to struggle at high equivalence ratios, which warrants further studies.

Previous studies have shown that water addition can significantly affect the LBV^9,34,35^ as well as the reaction pathways.^36^ This highlights the importance of experimentally measured LBV data for hydrous ethanol/air flames, particularly for developing accurate correlations under high water content conditions. However, experimental data in the literature remain limited, especially at elevated pressures and temperatures relevant to engine operation. van Treek et al.^37^ experimentally investigated the effects of water addition on the LBV of aqueous ethanol at atmospheric pressure. An empirical correlation of LBV with respect to the water fraction is used to model the impact of water addition. However, the influence of water addition at high pressure was not discussed. Outwardly expanding spherical flames with a schlieren imaging system were used by Liang et al.^38^ to measure the LBV of ethanol/air flames with water addition at 0.1 MPa and 383 K. Numerical simulations were also conducted to investigate the role of water addition in ethanol/air flames. While useful information about hydrous ethanol/air flames was provided, the temperature and pressure ranges studied were far from engine-relevant conditions, which necessitate further research on the effects of water addition on ethanol/air flames at elevated temperatures and pressures. Using the spherically expanding flame (pressure rise) method, Hinton et al.^17^ measured the LBV of hydrous ethanol/air flames at elevated initial temperatures of 380 and 450 K and pressures of 2 and 4 bar. LBV at high pressure was provided by a correlation, but significant scatter exists under fuel-rich and high dilution conditions. A similar experimental study conducted by Garzón Lama et al.^39^ presented the LBV of hydrous ethanol at an elevated pressure of 4 bar and a temperature of 450 K with a water volume fraction of up to 30%. Their results showed that the LBV is overpredicted by several mechanisms considered in their study. However, the LBV at higher temperatures and pressures is not available since the constant-pressure method was used. Additionally, the effects of water addition on the flame structure were not thoroughly addressed in these experiments. Motivated by these studies, this work expands on the existing correlation in the previous study^17^ to improve its performance at fuel-rich and high water content conditions and details the effects of water addition on ethanol/air flames at elevated pressure and temperature through numerical simulations.

To the authors’ knowledge, optimized LBV correlations for hydrous ethanol/air flames are scarce in the literature, especially at elevated temperatures and pressures relevant to engine operating conditions. Previous studies^37,40^ have shown that engines fueled by hydrous ethanol can operate stably with a water content up to 40%. Therefore, in this study, a water content of 40% by volume is considered, even though it may exceed that used in practical engine applications. In addition, this work primarily focuses on the fundamental properties of hydrous ethanol/air flames. Extended data at high water content are necessary for validating the correlations and ensuring the reliability of the correlations. Furthermore, although the role of water addition in ethanol/air flames has been discussed in previous studies, the temperature and pressure ranges considered are generally far from engine-relevant conditions. This study focuses on the effects of a high water content (up to 40%) under high-temperature and high-pressure conditions, which are more representative of practical applications.

Given the above-mentioned considerations, this study aims to develop a novel correlation for LBV calculation and reveal the effects of water addition on the LBV and flame structure of a hydrous ethanol/air mixture with high water content at elevated temperature and pressure. The new correlation is developed based on LBV data reported in previous work,^17^ where a 14-term correlation was used. This LBV data set covers wide ranges of initial temperature, pressure, and water content, making it suitable for validating the proposed correlation. Additionally, owing to the constant-volume method, data at elevated temperatures and pressures are available. The remainder of this paper is structured as follows. The methodology section introduces the fitting procedure for the experimental data and numerical details. Then, the development and validation of the new correlation for LBV, and numerical analysis on hydrous ethanol/air flames, are detailed and discussed. Finally, the effects of water addition on ethanol/air flames are summarized.

## Correlation Fitting Procedure and Numerical Setup

### Experimental Data and Correlation Fitting Procedure

The LBV data used for the optimization of correlations is provided in the previous study.^17^ Two different approaches, the pressure rise method and the flame front imaging method, were employed, and their reconciliation was achieved.^17,33^ The initial temperatures and pressures considered in the experiments are *T*_*u*_ = 380 K, 450 K, and *P*_*u*_ = 2.0 bar, 4.0 bar.^17^ The volume fractions of water are *x* = 0, 20, and 40%, denoted by W0, W20, and W40, respectively. At the end of spherical flame propagation, the temperature and pressure of the unburnt gas increase significantly from their initial values, providing LBV data at elevated temperatures and pressures. For W40, the maximum temperature and pressure of the LBV data set are 624 K and 14.8 bar, respectively. Note that since the dependence of LBV on water content is nearly linear, experimental data at two water content values are sufficient for the correlation fitting process, as they cover a wide range of initial temperatures, pressures, and equivalence ratios. More details regarding the validated range of LBV data sets can be found in.^17,41^ The schematic of the experimental data processing is shown in Figure 1. The mixture is prepared based on Dalton’s law of partial pressures. The volume of liquid fuel required to produce the desired mixture after evaporation is calculated using the LabView program. Ignition is achieved through a pair of electrodes located at the center of the combustion bomb with a diameter of 160 mm and a viewing diameter of 40 mm. After successful ignition, a spherical ignition kernel formed and propagated outward. During the combustion process, the pressure in the bomb is measured using a pressure transducer. The pressure rise history in the bomb is recorded by a LabView interface and used in the *burnvel* program. A multizone model^42,43^ is used to obtain the LBV. The burning velocity is calculated in the *bvcalc* program, and its correlation is fitted in the *fitcorr* program. For the flame imaging method, the schlieren images during flame propagation are recorded by a high-speed camera. Then, the images are processed by the *BVImage* program and the *refreshbg* subprogram to obtain the flame radius as well as the flame speed. Details of the experimental setup and the data processing procedure can also be found in previous studies.^17,33,43,44^

**Figure 1:**
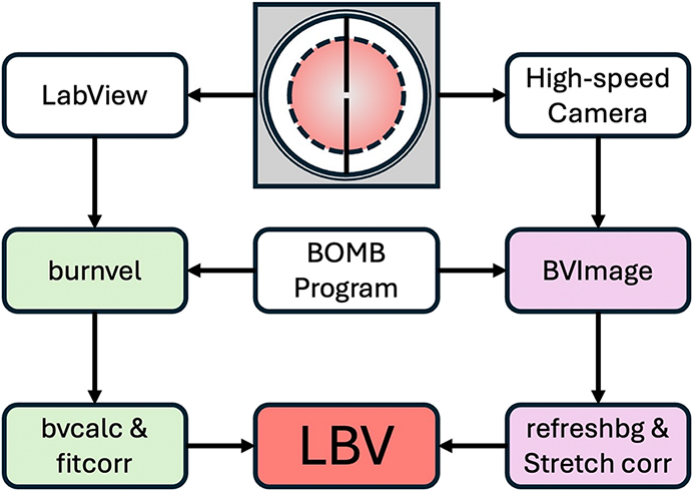
Schematic of experimental data processing. Green: pressure rise method; purple: flame front imaging method.

In previous studies,^17,33^ a 14-term correlation is fit to the LBV data measured using the pressure rise method:

$$S_{u}=S_{u,\mathrm{ref}}T^{\alpha }P^{\beta }F(x,\;\phi )$$ 1

where the reference speed *S*_*u*,ref_, temperature exponent α, and pressure exponent β only depend on equivalence ratio ϕ:

$$S_{u,\mathrm{ref}}=S_{u,0}+S_{u,1}(\phi -1)+S_{u,2}(\phi -1{)}^{2}+S_{u,3}(\phi -1{)}^{3}+S_{u,4}(\phi -1{)}^{4}$$ 2

$$\alpha =a_{0}+a_{1}\left(\phi -1\right)+a_{2}{\left(\phi -1\right)}^{2}$$ 3

$$\beta =b_{0}+b_{1}\left(\phi -1\right)+b_{2}{\left(\phi -1\right)}^{2}$$ 4

$$T=\frac{T_{u}}{298};\;P=\frac{P_{u}}{1.0}$$ 5

The dependence of LBV on water content *x* is modeled using a dilution function:^16,17^

$$F(x,\;\phi )=1-\mu_{1}x^{\mu_{2}+\mu_{3}(\phi -1)}$$ 6

The coefficients of the correlation are determined through a nonlinear optimization procedure. The error function, *f*, is defined as the sum of squares of the difference between the logarithm of the LBV calculated using the correlation and that measured in experiments:^43^

$$f=\sum (\log\;S_{u,\mathrm{corr}}-\log\;S_{u,\exp}{)}^{2}$$ 7

This error function is minimized to obtain the correlation coefficients. Specifically, the fitting procedure involved three sequential steps. First, using the LBV data in stoichiometric ethanol/air mixtures without water addition, three coefficients (*S*_*u*,0_, *a*_0_, and *b*_0_) are optimized. Second, all of the LBV data without water addition are used to determine the correlation coefficients relevant to *S*_*u*,ref_, α, and β. These coefficients are then held constant in the third step, where three coefficients associated with the dilution function are optimized using the LBV data with water addition.

Previous studies^17,45^ have shown that this 14-term correlation is good under a wide range of conditions. However, for the high water content (40%), this correlation from the pressure rise method shows scattered values compared to those derived from the flame front imaging method.^17^ Since this study focuses on the ethanol/air flames at elevated pressure and temperature, the correlation is optimized; a new correlation is proposed in the following section to better model the dependence of LBV on pressure and temperature under fuel-rich and high water dilution conditions.

### Numerical Method

The numerical simulation and flame structure analysis are performed using the open-source software Cantera.^46^ The ideal gas equation of state and the mixture-averaged transport model are adopted in the simulations. The radiation effects are not considered in this study. Since Soret diffusion has negligible effects for hydrocarbon fuels, even under engine-relevant conditions,^47^ it is neglected in this study. One-dimensional, adiabatic, unstretched, planar flames are simulated at various temperatures and pressures to obtain the LBV corresponding to the experimental conditions. The domain length in our simulations is 0.03 m. To ensure convergence, we use three refinement criteria based on grid spacing ratio, slope, and curvature: ratio = 3.0, slope = 0.07, and curve = 0.14. To quantify the effect of water addition on the structure of the hydrous ethanol/air flames, the spatial profiles of the major and minor species, temperature, and heat release rate (HRR) along the flow direction are extracted from one-dimensional free-propagating premixed flame simulations. The HRR is calculated as the sum of the volumetric HRRs from all of the elementary reactions.

To compare the experimental results with the simulations, several chemical mechanisms in the literature are used, as listed in Table 1. It is noted that in the study,^17^ the Olm mechanism^48^ was shown to agree well with the experimental data. Moreover, it was demonstrated^39^ that the predictions from the San Diego mechanism^49^ show good agreement with the experimental data. The Dryer mechanism^50^ is also used for comparison. Furthermore, another recently developed mechanism, FFCM2,^51^ is considered in the simulations, as this mechanism is validated over a wide range of thermodynamic conditions.

**Table 1:** Detailed Chemical Mechanisms for Ethanol Oxidation Considered in This Study

mechanism	year	species number	reaction number
Dryer^50^	2008	55	290
Olm^48^	2016	49	251
San Diego^49^	2016	58	270
FFCM2 ^51^	2023	96	1054

The correlation of LBV is crucial for the expression of LBV measurements using the constant-volume method. As discussed in the study,^17^ the pressure exponent can significantly affect the correlation at elevated temperature and pressure. To improve the performance of the LBV correlation, a series of simulations are first performed to evaluate the dependence of α and β on the equivalence ratio, pressure, and temperature.

## Results and Discussion

In order to develop the new correlation, we first examined the dependence of temperature and pressure exponents, α and β, on the thermodynamic states of the fresh mixture. Many studies^37,39^ have shown that a linear relationship is sufficient to model the dependence of LBV on diluent water fraction *x*. Therefore, eq 6 is not modified, and we focus on the temperature and pressure exponents α and β.

### Dependence of Temperature Exponent a on Equivalence Ratio and Pressure

The dependence of α on the equivalence ratio of the ethanol/air mixture has been discussed in several previous studies.^22,32^ It is found that a quadratic dependence of the temperature exponent on the equivalence ratio provides a better fit than a linear relationship. To verify this underwater addition, the dependence of α on the equivalence ratio ϕ at several initial pressures for the water content of 20% is shown in Figure 2. A minimum of α is observed around ϕ = 1.1 for various initial states. Moreover, both the FFCM2 and Olm mechanisms predict similar trends, which also agree well with previous studies.^22,32^ Therefore, adopting the quadratic formula with respect to ϕ in eq 3 can capture the nonmonotonic dependence of α on the equivalence ratio.

**Figure 2:**
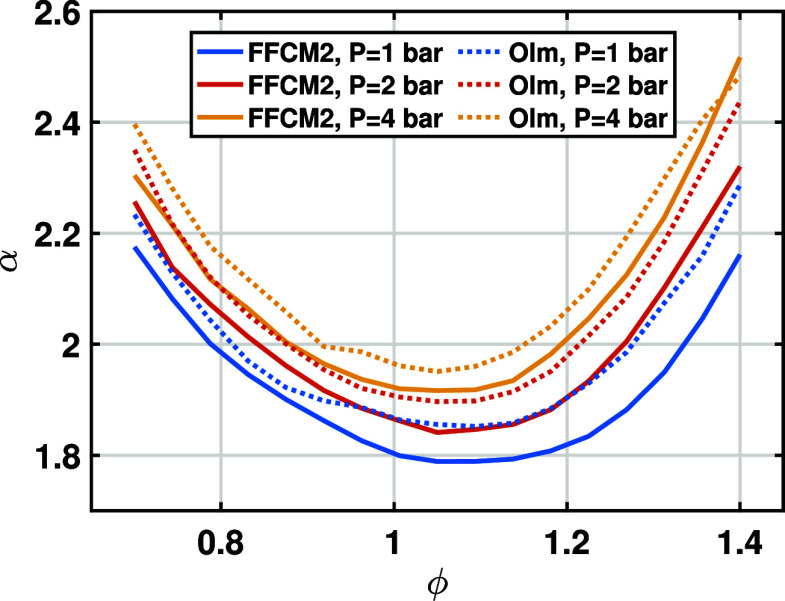
Dependence of temperature exponent α on the equivalence ratio ϕ for W20.

The relationship between α and pressure for various water contents and equivalence ratios is shown in Figure 3. It is seen that α increases slightly with the pressure for W0 shown in Figure 3a, demonstrating that the temperature exponent is not constant as the initial pressure changes. For W20 with 20% water addition in Figure 3b, α increases monotonically with pressure for ϕ = 0.7 and 1, while α calculated with the Olm mechanism exhibits a nonmonotonic feature for ϕ = 1.4. Nevertheless, the difference between the two mechanisms is not significant. In contrast, for W40 shown in Figure 3c, the predicted values of α from two mechanisms vary greatly for ϕ = 1.4, with a pronounced nonmonotonicity in the predictions from the FFCM2 mechanism. Moreover, α decreases significantly with increasing initial pressure for ϕ = 1.4, indicating that the pressure dependence of the temperature exponent α should be taken into account under fuel-rich conditions with a high water content.

**Figure 3:**
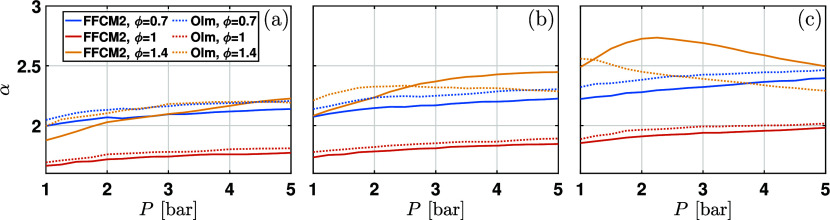
Dependence of temperature exponent α on pressure for (a) W0, (b) W20, and (c) W40.

Comparison in Figure 3 shows that high water content greatly affects the change of α with pressure, especially for the fuel-rich mixture shown in Figure 3c. Therefore, adding a cross-term to model the dependence of the temperature exponent α on the pressure in eq 3 is reasonable. Considering that the cross-term is the most pronounced for ϕ = 1.4 and W40 and the decreasing trend of α with pressure, an additional cross-term in the form of *a*_3_ *P*(ϕ – 1) is added to capture the effect of pressure on the temperature dependence of LBV.

### Dependence of Pressure Exponent β on Equivalence Ratio and Temperature

In addition to the temperature exponent α, the pressure exponent β is also affected by the equivalence ratio. Figure 4 shows the change of β with the equivalence ratio for initial temperatures of 380 and 450 K. As the equivalence ratio increases from 0.7 to 1.4, there exists a maximum of β, corresponding to ϕ ≈ 1.1. Similar trends are observed for W0 and W40. To avoid redundancy, they are not presented here. Therefore, a quadratic relationship between β and ϕ is applied to capture this feature.

**Figure 4:**
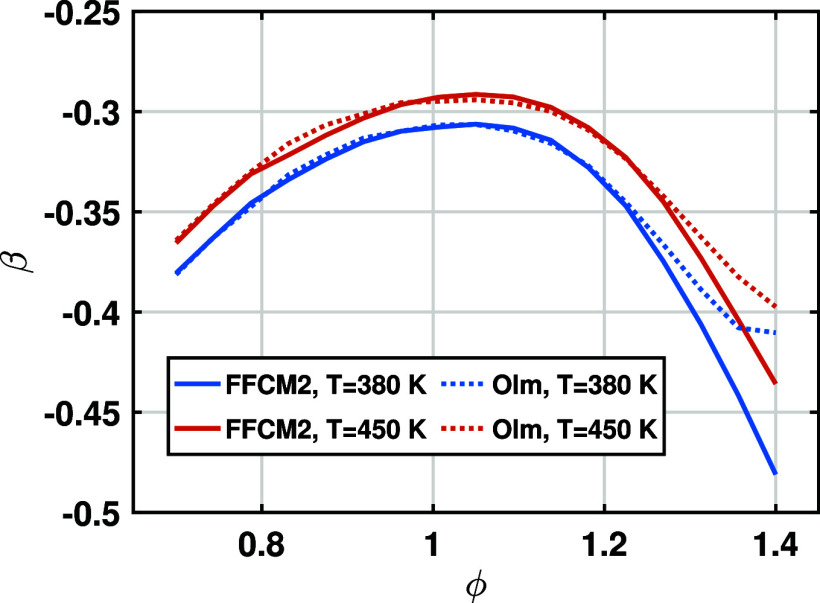
Dependence of pressure exponent β on equivalence ratio ϕ for *T*_0_ = 380 and 450 K and W20.

On the other hand, the pressure exponent β is also affected by the initial temperature. As shown in Figure 5, the value of β changes slightly with the initial temperature, regardless of the equivalence ratio. For ϕ = 0.7 and 1, the value of β shows a decreasing trend as the water content increases. Both mechanisms gave consistent results. However, for the fuel-rich mixture at ϕ = 1.4, different trends of β are observed. The β predicted by the FFCM2 mechanism decreases with water content, while that predicted by the Olm mechanism increases slightly. Nevertheless, both predictions show a minimal change over the equivalence ratio range considered. Although this feature can be captured by adding a linear cross-term, *b*_3_ *T* in eq 4, eq 4 is not modified since the change of β with temperature is slight.

**Figure 5:**
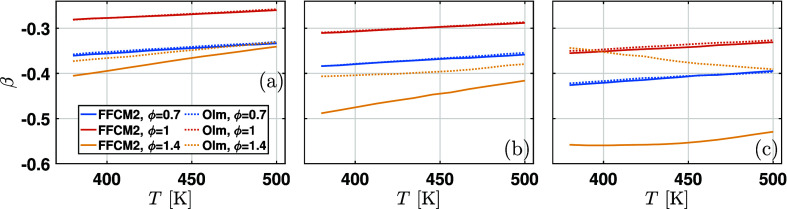
Dependence of β on temperature *T* for (a) W0, (b) W20, and (c) W40.

### Correlations of LBV

Based on the analysis of temperature and pressure exponents, a new correlation is proposed in this study. The pressure exponent eq 4, the dilution function eq 6 are used in eq 1 without modification. The optimized expressions of *S*_*u*,*ref*_ and α used in the new correlation are (where the modifications in this work are shown in **bold font**):

$$S_{u,\mathrm{ref}}=S_{u,0}+S_{u,1}(\phi -1)+S_{u,2}(\phi -1{)}^{2}+S_{u,3}(\phi -1{)}^{3}+S_{u,4}(\phi -1{)}^{4}+S_{u,5}(\phi -1{)}^{5}$$ 8

$$\alpha =a_{0}+a_{1}(\phi -1)+a_{2}(\phi -1{)}^{2}+a_{3}P(\phi -1)$$ 9

Compared with eq 2, a high-order term, *S*_*u*,5_(ϕ – 1)^5^, is introduced in eq 8. In addition, a cross-term, *a*_3_ *P*(ϕ – 1), is added in eq 9. This forms a 16-term correlation. The LBV data measured using the pressure rise method are used to obtain the fitting coefficients through a least-squares minimization routine in the *fitcorr* program, as illustrated in Figure 1.

For the hydrous ethanol/air flame, the coefficients of the 16-term correlation are shown in Table 2. Note that the new correlation aims to improve the LBV calculation of fuel-rich mixtures at elevated pressure with a high water content. Therefore, the value of *a*_3_ is expected to be small at low pressure. This is confirmed by the small fitting coefficient of *a*_3_ in Table 2.

**Table 2:** Coefficients of the 16-Term Correlation of the Laminar Burning Velocity

*S*_*u*,0_	*S*_*u*,1_	*S*_*u*,2_	*S*_*u*,3_	*S*_*u*,4_	*S*_*u*,5_
35.421	21.376	–108.116	–133.899	–96.977	697.792

*a*_0_	*a*_1_	*a*_2_	*a*_3_
1.917	–0.0563	2.292	–0.0559

*b*_0_	*b*_1_	*b*_2_
–0.254	0.288	–0.655

μ_0_	μ_1_	μ_2_
1.534	1.451	–0.315

To evaluate the performance of this new correlation, the root-mean-square error (RMSE) ( $\sqrt{f/N}$ , where *N* is the number of LBV data points) is calculated. Different correlations of LBV, including a 14-term correlation from^17^ (Hinton14), a 15-term correlation (HO15) by adding a high-order term (eq 8) to Hinton14, a 15-term correlation (CR15) by adding a cross-term (eq 9) to Hinton14, and the newly developed 16-term correlation (CRHO16) by adding both the high-order term and the cross-term to Hinton14, are compared. The RMSE values for all data points used for fitting, as well as for selected points at ϕ > 1.1 where a large discrepancy exists, are listed in Table 3.

**Table 3:** RMSE for Various Correlations^a^

correlations	Hinton14	HO15	CR15	CRHO16
RMSE	0.1545	0.1058	0.1583	0.1289
RMSE (ϕ > 1.1)	0.0417	0.0416	0.0410	0.0409

a The CRHO16 correlation is proposed in this study.

It is seen from Table 3 that the RMSE of CR15 is slightly larger than that of Hinton14. Therefore, adding a new term or degree of freedom does not always reduce the fitting error. This is reasonable since the form of correlation is a source of errors. A good correlation can reduce the error introduced by its form. The CRHO16 correlation proposed in this study exhibits a smaller RMSE compared to that of the Hinton14 correlation, demonstrating that the new correlation performs better than the original correlation.

The HO15 correlation is found to have the smallest RMSE among the four correlations examined in Table 3. However, for ϕ > 1.1, the RMSE is similar to the original correlation, Hinton14. This implies that adding a high-order term alone cannot improve the correlation for fuel-rich mixtures, where a large discrepancy between the correlation and experimental data exists. In contrast, both CR15 and CRHO16 show pronounced improvements with smaller RMSE for ϕ > 1.1, demonstrating that the cross term is important for the LBV of fuel-rich mixtures. This is consistent with the numerical results shown in Figure 3.

The new correlation is further validated against the LBV data measured by using the flame front imaging method. Note that consistency and reconciliation of the two methods are achieved in the previous study.^33^ Therefore, the comparison is reasonable. Figures 6 and 7 show the LBV measured from the pressure rise method as well as the flame front imaging method. It is found that both methods give consistent LBVs. All correlations are found to agree very well with the LBV measured using the flame front imaging method under fuel-lean and near-stoichiometric conditions. The discrepancy appears at fuel-rich conditions (ϕ > 1.1) and increases with the water content. It is clear that adding the cross-term or the high-order term solely leads to limited improvement of the LBV under fuel-rich conditions. Using the cross-term and the high-order term simultaneously results in the best agreement with the LBV from the flame front imaging method, especially when the water content is high. Therefore, this new 16-term correlation can improve the performance for fuel-rich mixtures with a high water content while maintaining the accuracy of the LBV calculation for fuel-lean ethanol/air mixtures.

**Figure 6:**
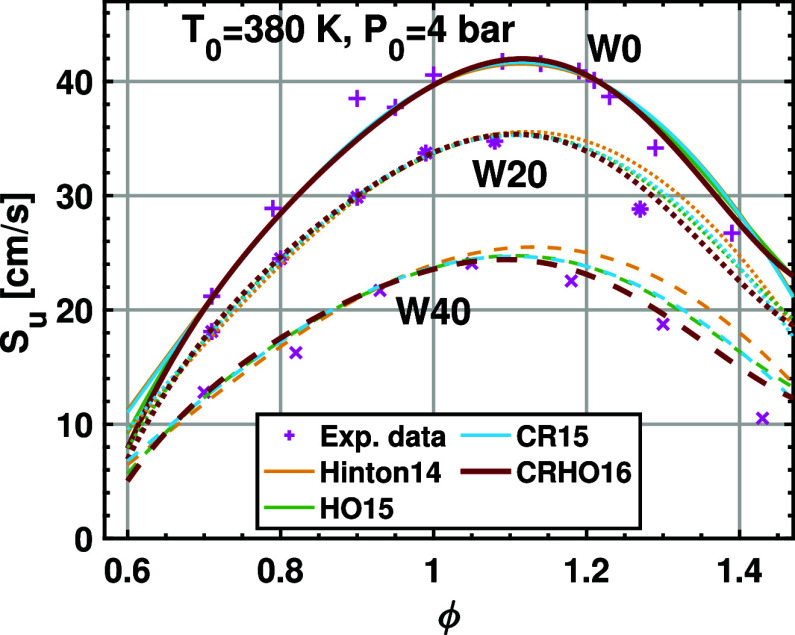
Laminar burning velocity data for hydrous ethanol/air mixtures at 4 bar and 380 K; W0, W20, and W40 are ethanol–water blends containing 0%, 20% v/v, and 40% v/v of water. The scatters are from flame front imaging, and the curves are from the correlations.

**Figure 7:**
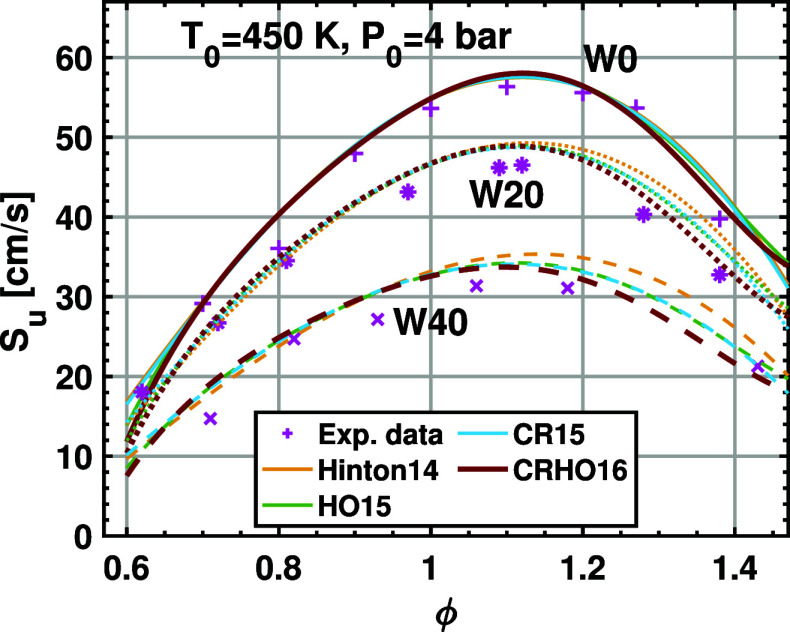
Laminar burning velocity data for hydrous ethanol/air mixtures at 4 bar and 450 K; W0, W20, and W40 are ethanol–water blends containing 0%, 20% v/v, and 40% v/v of water. The scatters are from flame front imaging, and the curves are from the correlations.

The LBV data shown in Figures 6 and 7 clearly demonstrate that the water addition leads to a significant decrease in flame speed. This is reasonable since the water addition increases the heat capacity of the mixture and thereby reduces the adiabatic flame temperature, which inhibits the chemical reaction. Moreover, water as a dilution can reduce the concentration of ethanol and oxygen, as well as the reactivity of the mixture. As expected, increasing the initial temperature of the mixture results in a higher LBV since the chemical reaction is significantly promoted by higher temperature. Therefore, water addition has significant effects on the reduction of the LBV.

The new 16-term correlation is compared with the data from the literature. Since the LBV data of the hydrous ethanol/air flame at elevated temperature and pressure are scarce in the literature, the LBV is extrapolated to lower temperature and pressure through the newly developed correlation. Figure 8 shows the LBV from the 16-term correlation and the literature^22,37,52–55^ at *T*_0_ = 358 K and *P*_0_ = 1 bar. The new correlation is found to agree well with the literature data under fuel-lean conditions. However, under fuel-rich conditions, the literature data are larger than the LBV values given by the correlation. This might result from the fact that fuel-rich data used for correlation fitting is less than the fuel-lean data, as mentioned in the previous study.^17^ Nevertheless, the new correlation agrees reasonably well with previous measurements^52,53^ at fuel-rich conditions.

**Figure 8:**
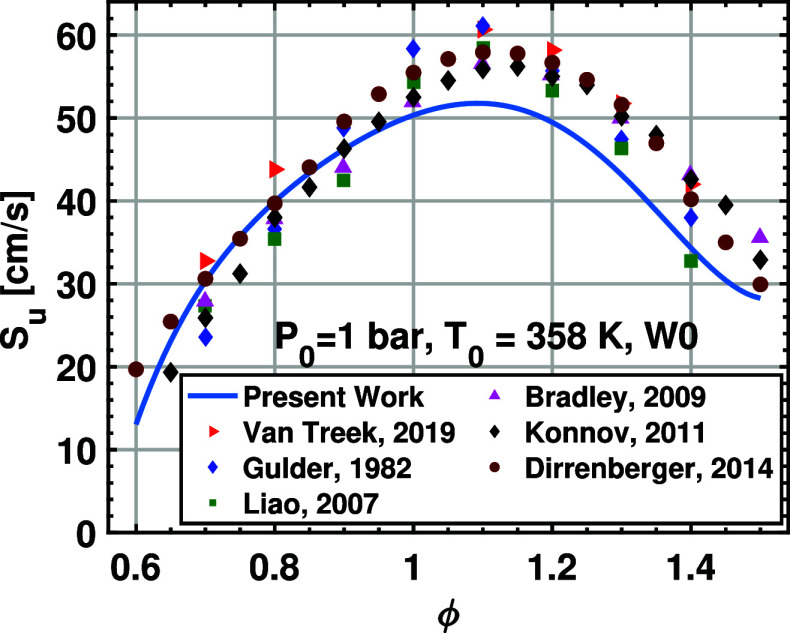
Laminar burning velocity data for ethanol/air mixtures at 1 bar and 358 K. The scatters are from several published data, and the curves are from the 16-term correlation.

At elevated pressure, the correlation also provides a reasonable prediction of the LBV for the ethanol/air mixture. As shown in Figure 9, for a wide range of initial pressure from *P*_0_ = 5 to 14 bar, the correlation of this study gives consistent LBV values with the early experimental data published in the previous study.^54^ In addition, our new correlation agrees well with the experimental data^24^ at *T*_0_ = 373 K and *P*_0_ = 10 bar. Therefore, the performance of our correlation is good at an elevated temperature and pressure. Note that the new correlation is not validated at extremely high pressures above 20 bar, which are far beyond the valid conditions of the available LBV data. The reason is 2-fold: first, experimental LBV data above 20 bar are not available; second, validation against LBV predictions from detailed chemical mechanisms is not reliable, as existing mechanisms are not well validated under such high pressures.

**Figure 9:**
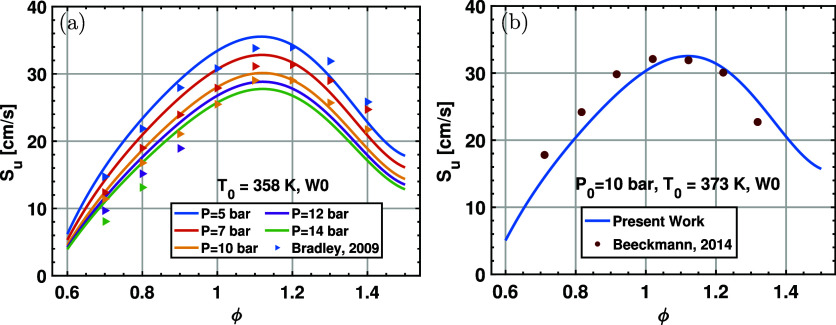
Laminar burning velocity data and a new correlation for ethanol/air mixtures at elevated temperatures and pressures. The scatters are from published data, and the curves are from the 16-term correlations.

Additionally, for the hydrous ethanol/air mixture, the correlation also agrees well with the literature data,^39^ as shown in Figure 10. At 450 K and 4 bar, LBV calculated using the new correlation is consistent with the experimental data over a wide range of water volume fraction. This also demonstrates that the new correlation is able to predict the LBV of a hydrous ethanol/air mixture with high water content at high pressure and temperature.

**Figure 10:**
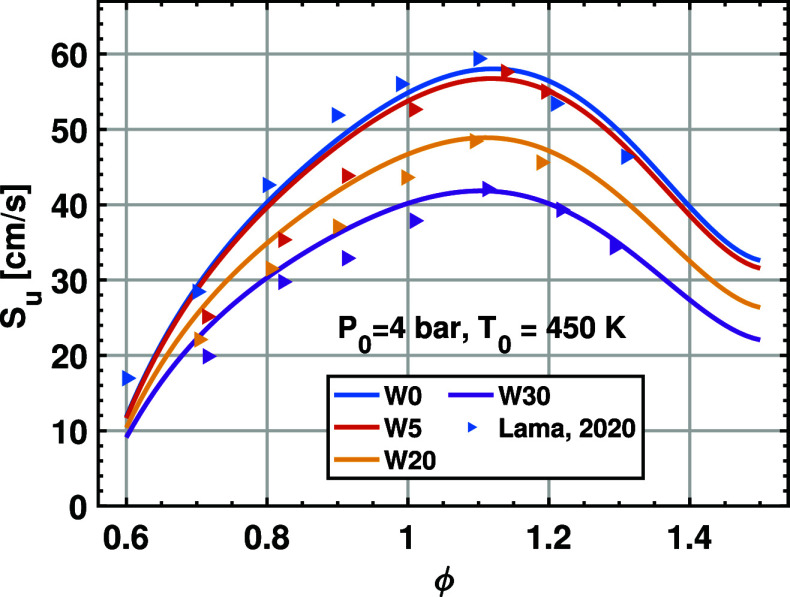
Laminar burning velocity data and new correlation for hydrous ethanol/air mixtures at 4 bar and 450 K. The scatters are from published data, and the curves are from the 16-term correlation proposed in this study.

Furthermore, the new correlation of LBV is compared to simulation results using various mechanisms, as shown in Figure 11. Generally, the correlation calculation agrees well with numerical results. For ϕ < 1.1, the predicted LBV using the FFCM2 mechanism agrees very well with the new correlation. Specifically, for W0 without water addition, the LBV predicted by FFCM2 and Olm mechanisms agrees very well with the correlation; the LBV predicted by Dryer and San Diego mechanisms is consistent with the correlation at fuel-rich conditions, while larger values of the LBV are observed for fuel-lean mixtures. As the water content increases to W20 and W40, a discrepancy in LBV between predictions from Olm and FFCM2 mechanisms and the correlation is observed at ϕ > 1.1. This phenomenon was also observed in previous studies.^17,37^ Therefore, there is a need for future studies to improve the accuracy of LBV prediction from detailed chemical mechanisms under fuel-rich conditions.

**Figure 11:**
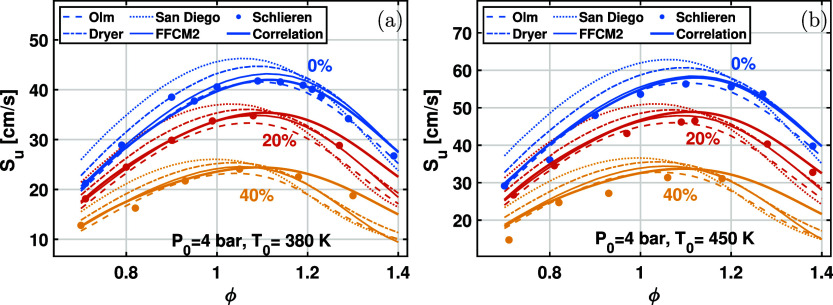
Laminar burning velocity data for ethanol/air mixtures at *P*_0_ = 4 bar and (a) *T*_0_ = 380 K and (b) *T*_0_ = 450 K. The scatters are from flame front imaging data, and the solid curves are from the correlations. Chemical mechanisms used in the simulations are listed in Table 1.

### Isolated Effect of Water Addition

Water addition affects the LBV in various ways. First, adding water to the fresh mixture leads to a reduction in the reactant concentration and thus the chemical reaction rate. This dilution effect can inhibit the LBV. Second, the addition of water changes the heat capacity of the mixture as well as the transport properties. These thermophysical properties also affect LBV. Meanwhile, water has a chemical effect on LBV since it participates in the chemical reactions directly as a reactant or product and indirectly as a third-body collider.

To isolate the dilution effects due to the addition of water, an additional case is considered in which 40% water in the hydrous ethanol/air flame (W40) is replaced with an equivalent mole fraction of nitrogen (W40–N2). By substituting water with an inert gas (nitrogen), the dilution effect can be isolated through comparison between the cases with and without nitrogen addition.^25,56^ The LBV data for W0 and W40–N2 are shown in Figure 12. The dilution effect is found to have a great contribution to the decrease in LBV. With 40% addition of dilution, the peak of LBV decreases from 60 to 40 cm/s.

**Figure 12:**
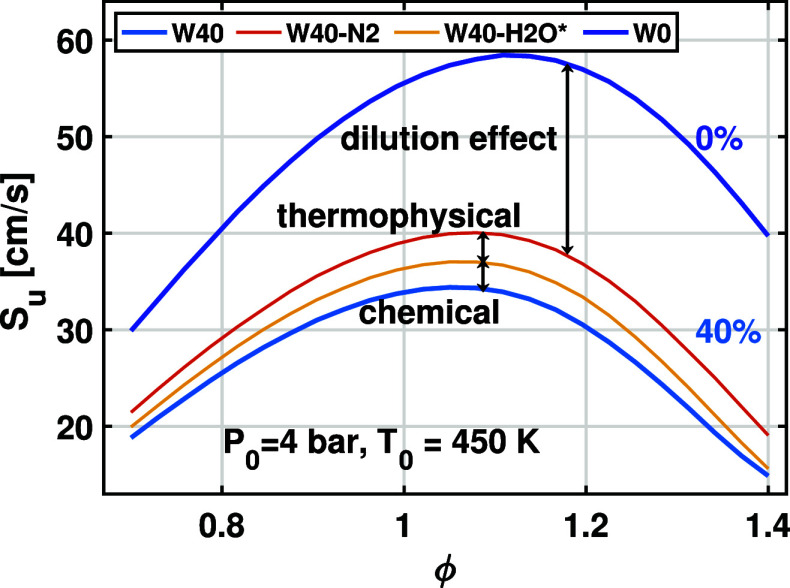
Isolated effect of chemical, dilution, and thermodynamic on laminar burning velocity for W40 at *P*_0_ = 4 bar and *T*_0_ = 450 K.

The thermophysical and chemical effects caused by the addition of water are isolated by introducing a case using the fictitious diluent (W40–H2O*).^25^ The fictitious diluent, H2O*, has the same thermal and transport properties as water, but the chemical properties are the same as those of nitrogen. This is achieved by artificially modifying the Cantera input files. Similar methods were used in previous studies.^25,38,57^ As shown in Figure 12, the LBV of W40–H2O* is consistently smaller than that of W40–N2, indicating that the thermophysical effect of water leads to a reduction in the flame speed. This is reasonable since the heat capacity of water is higher than that of nitrogen. The temperature profiles shown in Figure 17 also demonstrate this effect.

The chemical effect of water addition can be addressed by the comparison between W40 and W40–H2O*. It is seen from Figure 12 that neglecting the chemical reaction due to water addition leads to an increase in the LBV. The increment is comparable to the difference between cases W40–N2 and W40–H2O*, indicating that the thermophysical and chemical effects have similar contributions to the decrease in LBV.

In summary, the thermophysical and chemical effects due to water addition have a minor and similar contribution to the reduction of LBV, whereas the single largest factor in reducing LBV is its dilution effect. To better understand the chemical and thermophysical effects, we conducted chemical sensitivity analysis and flame structure analysis on hydrous ethanol/air flames in the following sections.

### Sensitivity Analysis

Feng et al.^36^ showed that the reaction pathways can be affected by the water addition. To investigate the effect of water addition on the chemical reaction of ethanol, the sensitivity of the reaction coefficients is examined. Note that a positive value of the sensitivity coefficient indicates that the reaction enhances the LBV and vice versa. Consistent with the other sections, the FFCM2 mechanism is used for the sensitivity analysis. The flame speed sensitivity is evaluated using the adjoint method.

Figure 13a shows the 15 most important reactions with large (absolute) sensitivity coefficients at ϕ = 0.7 for W0 and W40. Since the coefficient of H + O_2_ = O + OH is significantly larger than those of other reactions, it is divided by a factor of 2 for better visualization, as indicated by × 2 in the figure. It is seen that the sensitivity coefficients do not change much with the water content. The chain-branching reaction H + O_2_ = O + OH and the chain-carrying reaction CO + OH = CO_2_ + H have the largest contributions to LBV. These two reactions are widely recognized as fundamental in the combustion of hydrocarbon fuels.^10^ For the reaction with a large negative sensitivity coefficient, H + O_2_ (+M) = HO_2_ (+M), its importance increases as the water content increases from 0 to 40%. Since the active H radical is consumed while the inactive HO_2_ is produced, this reaction strongly inhibits the LBV for W40. Similar phenomena can be found for ϕ = 1.0, shown in Figure 13b.

**Figure 13:**
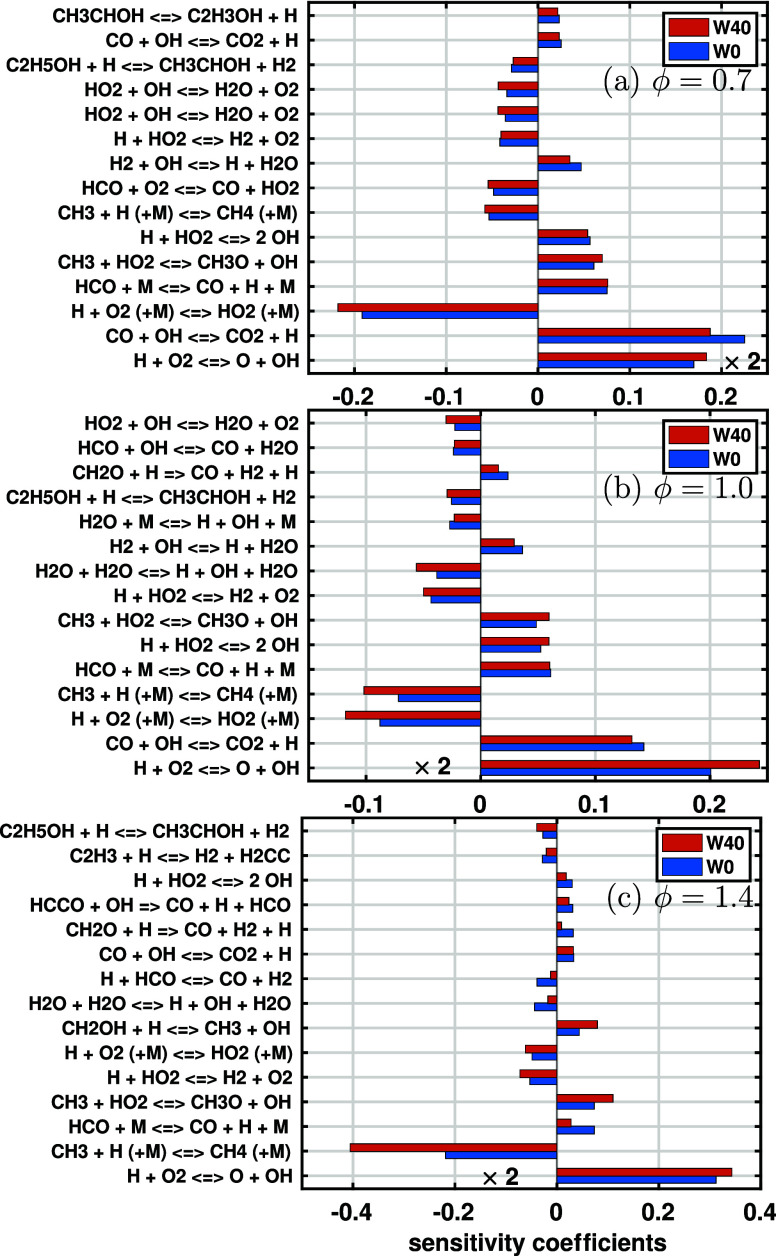
Sensitivity analysis of the LBV of the ethanol/air flame for W0 and W40 at *P*_0_ = 4 bar and *T*_0_ = 450 K. The equivalence ratios are (a) ϕ = 0.7, (b) ϕ = 1.0, and (c) ϕ = 1.4.

For ϕ = 1.4, a pronounced difference from the fuel-lean and stoichiometric conditions is that of reaction R100: CH_3_ + H (+M) = CH_4_ (+M) becomes very important at a high water content. As shown in Figure 13c, the sensitivity coefficient for W40 is almost twice that of W0, demonstrating that this reaction significantly inhibits the LBV of the ethanol/air mixture with water addition. This may be a potential reason for the large discrepancy in LBV between the correlation and the predicted values from different mechanisms, indicating the need for further studies on the oxidation of hydrous ethanol under fuel-rich conditions. To reveal the role of radicals on flame characteristics, we need further study of the flame structure.

### Flame Structure

In order to interpret the effects of water addition on ethanol/air flames, the laminar flame structure for several water contents and equivalence ratios is presented in Figure 14. Since the prediction using the FFCM2 mechanism is in good agreement with the experimental data, it is used to calculate the flame structure. The mole fraction profiles of several key species for fuel-lean ethanol-air flames are shown in Figure 14a. The flame front (defined as the location of the maximum gradient of the flame temperature) is adjusted to *x* = 0 to enable a clearer comparison of flame structures. It is seen that as the water content increases, the concentration of water in the burnt gas zone also increases. However, the mole fraction of CO_2_ decreases slightly with the water content. For a stoichiometric ethanol/air flame shown in Figure 14b, a higher water content leads to a lower mole fraction of CO. The mole fraction of CO_2_ in the burnt gas zone is almost unaffected by the water content. Similar trends are observed for the fuel-rich ethanol/air flame shown in Figure 14c.

**Figure 14:**
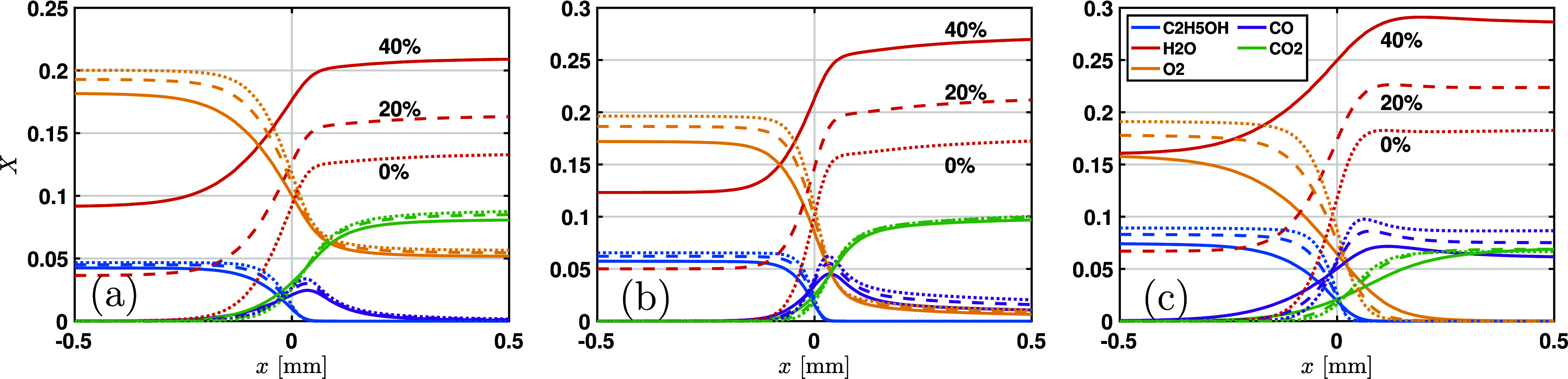
Flame structure for (a) ϕ = 0.7, (b) ϕ = 1.0, and (c) ϕ = 1.4 at *P*_0_ = 4 bar and *T*_0_ = 450 K. The FFCM2 mechanism is used for simulations.

Additionally, the water content also affects the concentration of minor species, such as OH and H radicals, as shown in Figure 15. For all equivalence ratios, the mole fraction of OH and H decreases with water content. Since OH and H are active species and are largely relevant to the LBV, it is clear that water addition leads to a significant reduction in active species and thus inhibits the LBV. Therefore, water addition has chemical effects on the flame structure as well as on the LBV.

**Figure 15:**
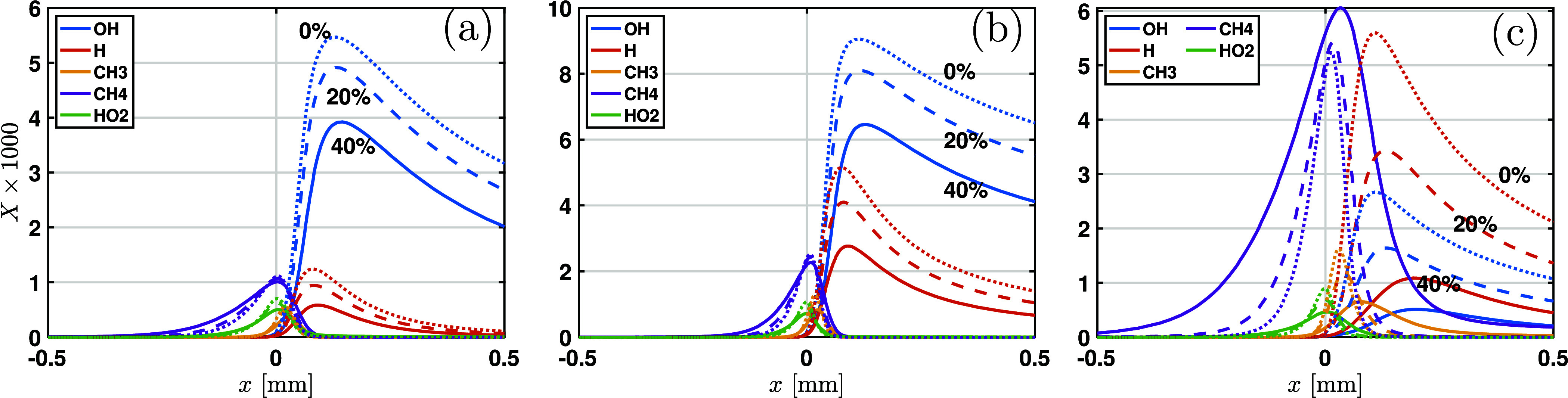
Flame structure for (a) ϕ = 0.7, (b) ϕ = 1.0, and (c) ϕ = 1.4 at *P*_0_ = 4 bar and *T*_0_ = 450 K. The FFCM2 mechanism is used for simulations.

It is interesting to note that the concentration of CH_4_ increases with the water content for ϕ = 1.4, which is different from the trends for ϕ = 0.7 and ϕ = 1.0. This is consistent with the sensitivity analysis shown in Figure 13. For ϕ = 1.4, the sensitivity coefficient relevant to the formation of CH_4_ increases significantly with the water content.

The sensitivity analysis shows that some reactions related to CH_4_ are significantly affected by water addition. To understand the role of these reactions, reaction rate distributions along the flow direction are analyzed. Key reactions with the five highest CH_4_ reaction rates are shown in Figure 16. It is clear that as the water content increases, the net rate for each reaction in the figure decreases substantially. Additionally, the net reaction rate exhibits an increasing trend with ϕ. The reaction R100: CH_3_ + H (+M) = CH_4_ (+M) is the most important reaction relevant to CH_4_ production, which is consistent with the results of the sensitivity analysis. For ϕ = 1.4, the consumption of CH_4_ through R142: CH_4_ + H = CH_3_ + H_2_ showed the largest reaction rate. The peak of the net rate of R142 and R100 is very close. Moreover, other reactions with a large rate, R144 and R143, are pathways for CH_4_ consumption. Therefore, CH_4_ formation through R100 largely influences subsequent consumption via R142, R144, and R143, explaining the results of the sensitivity analysis in Figure 13c.

**Figure 16:**
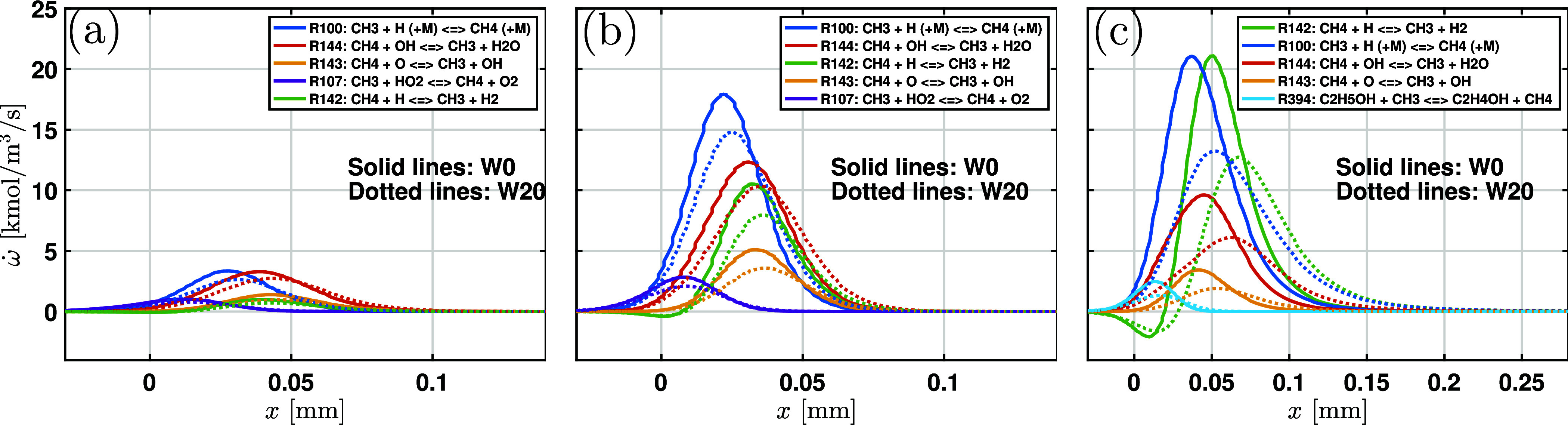
Profiles of the net reaction rate relevant to CH_4_ for (a) ϕ = 0.7, (b) ϕ = 1.0, and (c) ϕ = 1.4 at *P*_0_ = 4 bar and *T*_0_ = 450 K. The FFCM2 mechanism is used for simulations.

Adding water in ethanol also affects the physical properties of the mixture, since the specific heat capacity increases due to the addition of water. The temperature profiles shown in Figure 17 demonstrate that the water addition leads to a decrease in burnt gas temperature. This thermal effect can suppress the LBV. The water addition also significantly reduces the peak value of the HRR, especially under fuel-rich conditions of ϕ = 1.4. Specifically, at ϕ = 0.7, the peak of HRR decreases from 2.7 × 10^10^ to 1.1 × 10^10^ W/m^3^ as the water content increases from W0 to W40, while at ϕ = 1.4, the peak of HRR decreases from 4 × 10^10^ to 0.7 × 10^10^ W/m^3^. Therefore, the water addition has a stronger influence on fuel-rich ethanol/air flames.

**Figure 17:**
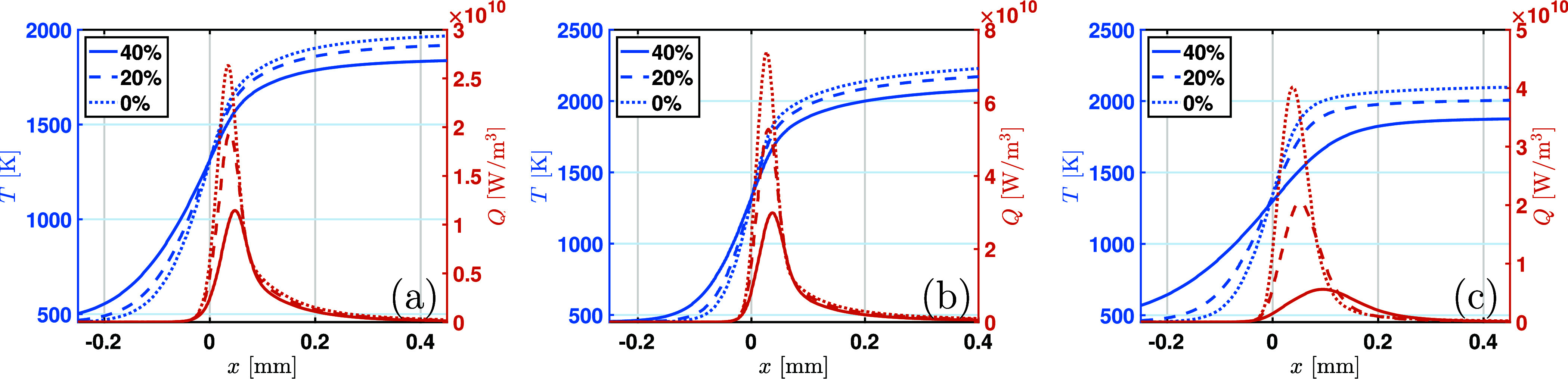
Flame structure for (a) ϕ = 0.7, (b) ϕ = 1.0, and (c) ϕ = 1.4 at *P*_0_ = 4 bar and *T*_0_ = 450 K. The FFCM2 mechanism is used for simulations.

To quantify the impact of dilution, chemical and thermophysical factors introduced by water addition on flame structure, the temperature and HRR profiles for W0, W40–N2, W40–H2O*, and W40 are compared, as shown in Figure 18. The dilution effect, represented by the difference between W0 and W40–N2, is the primary factor in reducing both the burnt gas temperature and the peak HRR. The thermophysical effect, indicated by the difference between W40–N2 and W40–H2O*, can also lower both the burnt gas temperature and the peak HRR, but its magnitude is much smaller than that of the dilution effect. The difference between W40–H2O* and W40 highlights the role of the chemical effect, which only lowers the HRR while leaving the burnt gas temperature essentially unchanged. Therefore, both dilution and thermophysical factors reduce LBV by lowering the combustion temperature and HRR, with the primary contribution from dilution. The chemical effect is minor, and the burnt gas temperature is largely unaffected by it.

**Figure 18:**
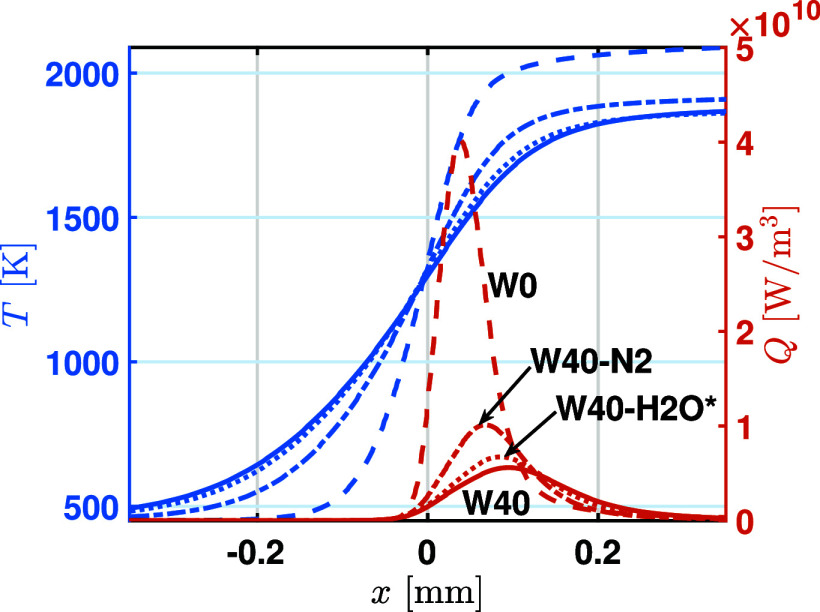
Isolated effect of chemical, dilution, and thermodynamic on flame temperature and heat release rate for W40 at ϕ = 1.4, *P*_0_ = 4 bar, and *T*_0_ = 450 K.

## Conclusions

In this study, the effects of water addition on the structure and propagation speed of ethanol/air flames with a high water content at elevated temperatures and pressures are analyzed through simulations. Based on the numerical results, a novel correlation for the LBV of hydrous ethanol/air flames is proposed and validated against various experimental data and numerical predictions. The LBV calculated using the new correlation based on the pressure rise method is consistent with the flame front imaging method for high water content up to 40% volume fraction, providing a reliable method for LBV modeling under engine-relevant conditions.

Through numerical simulations with well-validated chemical mechanisms, the dependence of temperature and pressure exponents on the equivalence ratio, initial pressure, and temperature is evaluated. The temperature exponent curve with respect to equivalence ratio is found to have a minimum around ϕ = 1.1, while the pressure exponent reaches a maximum. This agrees with previous studies, indicating that quadratic relationships between the temperature and pressure exponents with respect to the equivalence ratio are reasonable. In addition, the temperature exponent changes greatly at an elevated pressure for fuel-rich ethanol/air mixtures with high water content. This highlights the necessity of introducing a cross-term to capture the dependence of the temperature exponent on the pressure. In contrast, the dependence of the pressure exponent on temperature is weak, which is not important for the correlations.

Based on numerical analysis, a 16-term correlation of the LBV is proposed by taking into account the high-order term and the cross term. The LBV predicted by the new correlation is consistent with that of the flame front imaging method at high temperatures and pressures, especially for fuel-rich mixtures with a high water content. The error analysis demonstrates that adding both the high-order term and cross term results in an improvement in the correlation. The cross term plays a key role in reducing the error under fuel-rich conditions. Good agreement with the experimental literature data over a wide range of temperatures and pressures is achieved.

Both experimental data and simulations show that the LBV of the ethanol/air mixture is significantly reduced as the water content increases. A sensitivity analysis shows that the dominant reactions change for fuel-rich mixtures, and some important reactions become sensitive to water addition, which could be the reason for the discrepancy in LBV between experimental measurements and numerical predictions. Flame structure analysis shows that both the chemical effects and thermal effects of water addition lead to a reduction in LBV. Through a fictitious diluent gas method, the dilution, thermophysical, and chemical effects are isolated. The dilution effect is found to be the largest factor in reducing the LBV. The thermophysical and chemical effects are relatively minor.

The new correlation is essential for LBV calculations at high water content and elevated temperatures and pressures as well as simulations of ethanol combustion under engine-relevant conditions. Additionally, this study details a procedure to reduce the error of the correlation form, providing a generalized approach for optimizing the LBV correlations. In future studies, this correlation can be applied to other fuels to improve the modeling of the LBV.
